# Delay in Diagnosis of Addison's Disease: A Case Report and Literature Review

**DOI:** 10.1002/ccr3.73214

**Published:** 2026-07-27

**Authors:** Ishita Bhattacharya, Lisle Blackbourn, Manajyoti Yadav

**Affiliations:** ^1^ Department of Internal Medicine Indiana University School of Medicine Indianapolis Indiana USA; ^2^ Department of Internal Medicine University of Illinois College of Medicine at Peoria Peoria Illinois USA

**Keywords:** adrenal insufficiency, cognitive overload, delayed diagnosis, endocrine

## Abstract

Primary adrenal insufficiency is a rare disorder prone to delay in diagnosis after initial presentation. Multiple factors including a wide range of presentations, slow onset of symptoms, and other human factors may be contributing to challenges with the diagnosis.

## Graphical abstract

Graphic highlighting the timeline of ED visits and admissions for the case patient.

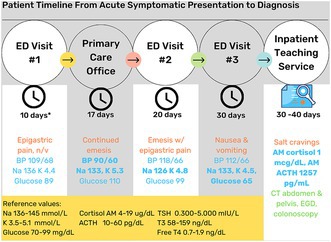

## 1 Introduction

Primary adrenal insufficiency (PAI), or Addison's disease, is a rare disease with a prevalence ranging from 40 to 140 cases per million inhabitants and an annual incidence ranging from 4 to 6 new cases per million inhabitants [1]. The broad clinical presentation of PAI makes it prone to misdiagnosis, including simple gastrointestinal illnesses and delay in diagnosis. Less than half of patients with adrenal insufficiency are diagnosed within 6 months of symptom presentation, with a fifth being diagnosed after 5 years [2]. Left undiagnosed, patients are at risk for adrenal crisis, which can lead to shock and death. The nonspecific presentation and ever‐increasing cognitive overload of physicians in busy emergency departments and offices pose diagnostic challenges for rare disorders such as PAI. We present a case of PAI highlighting the various clinical and non‐clinical challenges that contributed to the challenges in making the correct diagnosis.

## 2 Case History/Examination

A 55‐year‐old Caucasian female with a history of hypothyroidism initially presented to the emergency department (ED) with epigastric pain and a 10‐day history of nausea and non‐bilious, non‐bloody vomiting. Objective exam at that time was notable for a blood pressure of 109/58 mmHg and epigastric tenderness.

## 3 Differential Diagnosis, Investigations, and Treatment

Initial ED admission workup demonstrated no electrolyte abnormalities. Clinical diagnosis of gastritis was made, and she was discharged on ondansetron and antacids. She had 4–5 episodes of emesis the following week and followed up with her primary care physician. Further workup, including *H. pylori* stool sampling was ordered, and she was prescribed omeprazole and ondansetron. Labs revealed hyponatremia (133 mmol/L), hyperkalemia (5.3 mmol/L), and borderline hypotension (90/60 mmHg).

**FIGURE 1:**
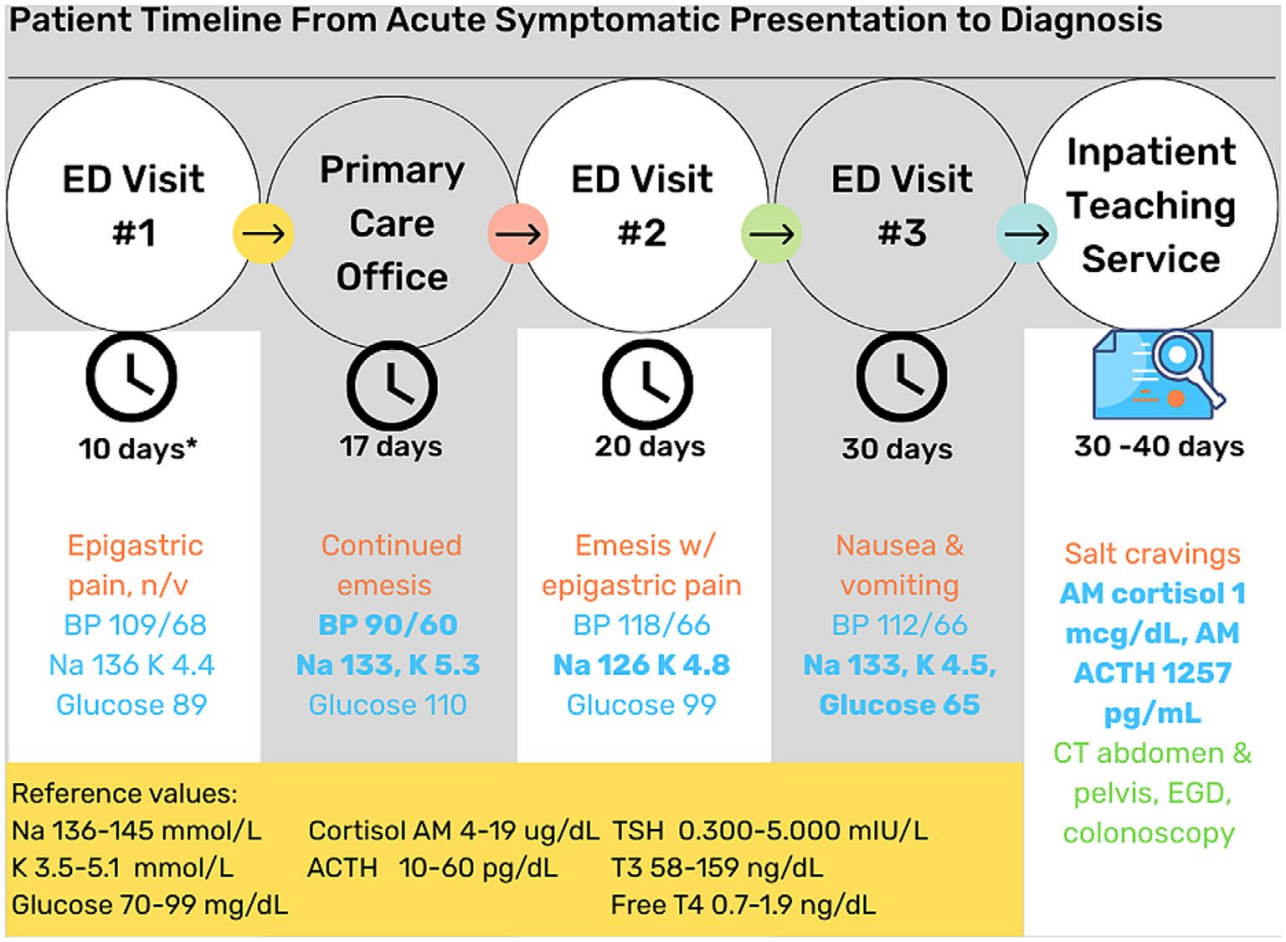
Graphic highlighting timeline of ED visits and admissions for the patient. Lab values for the patient and reference values also provided.

She presented to the ED 3 days later for ongoing emesis with epigastric pain. Sodium was again abnormal at 126 mmol/L and potassium normal at 4.8 mmol/L (refer to Figure 1 for reference values). Hyponatremia was attributed to recurrent emesis and the patient was discharged home from ED after symptomatic improvement with fluids. She again returned to the ED 10 days later for continued emesis with hyponatremia at 133 mmol/L, normal potassium at 4.5, and she was admitted to the hospital. The day after admission, the patient also had an episode of hypoglycemia at 65 mg/dL, which persisted the next day at 63 mg/dL despite fluid and food intake. The patient was admitted to an internal medicine teaching service team.

**FIGURE 2:**
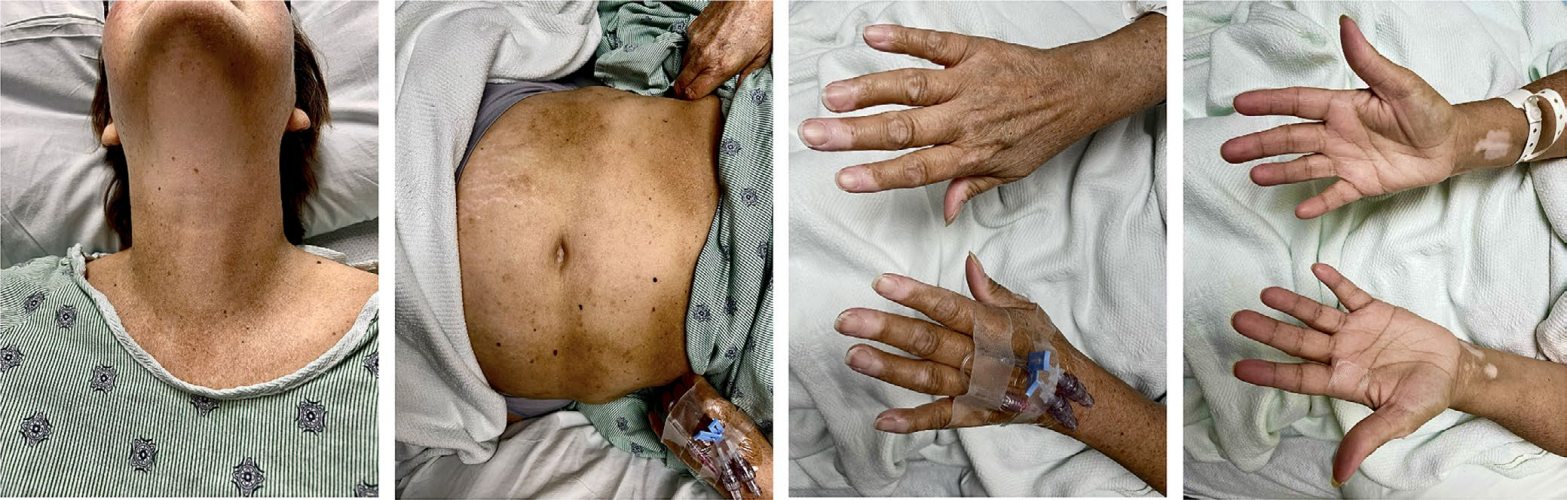
Hyperpigmentation around jaw line, outer neck, upper chest, and abdomen; and vitiligo patches on hands and wrists.

A thorough history and exam revealed vitiligo, darkening of the skin of her upper body (see Figure 2), and salt cravings that all began 2–3 months earlier. Prior documentation did not mention these findings. Additional labs included thyroid studies, with borderline low TSH 0.260 mIU/L, elevated T3 of 209 pmol/L, and T4 within normal limits. Diagnosis of hypothyroidism had been made 2–3 years prior, and she was on levothyroxine 100 mcg daily. The team ordered a CT abdomen and pelvis and consulted gastroenterology for an esophagogastroduodenoscopy and colonoscopy. These imaging tests and procedures did not have any findings suggestive of malignancy or an alternate diagnosis.

After initial testing, PAI workup was pursued which yielded very low AM cortisol (1 mcg/dL), very high AM adrenocorticotropic hormone (ACTH) (1257 pg/mL), and positive results for presence of 21‐hydroxylase antibodies. The patient was started on hydrocortisone 10 mg and fludrocortisone 20 mg daily.

### 3.1 Outcome and Follow‐Up

The patient showed significant improvement in symptoms within a few days of initiating treatment. The patient was discharged home with ongoing treatment and outpatient follow‐up (see Figure 1).

## 4 Discussion

PAI typically presents in females between the ages of 30 and 50; however, there have been a few reported cases of pediatric diagnoses of PAI [3]. Our patient's presentation may be considered a less common, later presentation due to her age, supporting the findings of Oelker [4]. Her case also illustrates how PAI can be a missed diagnosis, leading to multiple hospital admissions, excessive testing, adding costs, stress to the patient, and delay in treatment. Many factors contributed to this delay in diagnosis, including non‐specific symptoms, other similar or more common diagnoses, obscured physical exam findings, rarity of the disease, and likely the cognitive overload of providers in busy practices.

### 4.1 History and Physical

The findings of salt cravings, hyperpigmentation, vitiligo, hypothyroidism, and hypotension in our patient led to the suspicion of PAI. Hyperpigmentation was found to be the most common presenting symptom, with retrospective studies finding a prevalence of 86%–94% [5, 6]. Hyperpigmentation is most commonly noted at the palmar creases and of the mucosal membranes [7]. Our patient however, did not have mucosal involvement. Although a common finding in patients with PAI, hyperpigmentation is often missed by providers on physical exam. A cross‐sectional study found skin abnormalities to be one of the most common missed findings leading to delayed medical diagnoses [8]. Salt cravings are commonly associated with Addison's disease, although it is not a very common presenting symptom, with approximately 16% of patients reporting salt cravings [6]. People with autoimmune conditions, such as vitiligo, are more likely to develop other autoimmune conditions. Vitiligo was found in 10%–20% of patients with PAI. Zelissen, Bast, and Croughs found that 47% of patients with PAI had another autoimmune condition present at the time of diagnosis [9].

Stokland et al. found autoimmune thyroid disease to be the most common concomitant autoimmune disorder with PAI, with 48% of patients having both [10]. The association of autoimmune thyroid disease and PAI is characteristic of Autoimmune Polyendocrine Syndrome Type 2, or Schmidt's Syndrome. Our patient underwent autoimmune testing by her endocrinologist for Hashimoto's thyroiditis after her PAI diagnosis and had an elevated thyroid peroxidase antibody count of 3371 IU/mL, confirming concurring autoimmune hypothyroidism and Schmidt's syndrome.

Hypotension is also a common sign in PAI, with approximately 90% of patients presenting with hypotension [6]. Patients presenting with unexplained hypotension or persistent hypotension in the setting of fluid resuscitation should be further evaluated for possible PAI diagnosis.

### 4.2 Laboratory Studies

The duration of symptoms and the concurrent hyponatremia, hyperkalemia, and hypotension despite fluid resuscitation and treatment indicate potential adrenal insufficiency. While hyponatremia may be seen in emesis due to other etiologies, hyperkalemia is not expected. Normokalemia within the upper ranges of normal in the setting of multi‐episodic emesis may also indicate insufficiency, especially with previously recorded hyperkalemia. Saevik et al. found episodes of hyponatremia in 84% of patients and hyperkalemia in only a third of patients during hospital admissions prior to diagnosis [11].

### 4.3 Differential Diagnoses

The presentation of gastrointestinal symptoms of our patient led to the examination for several differential diagnoses, including gastritis and malignancy. Other differential diagnoses that are important to consider in the setting of recurrent nausea and vomiting include SIADH, anorexia nervosa, and secondary causes of adrenal insufficiency such as hypopituitarism or excessively rapid withdrawal of corticosteroid use [12]. When primary adrenal insufficiency is confirmed, etiology must be distinguished between infectious and autoimmune.

### 4.4 Cognitive Overload

Aside from the clinical challenges that led to delay in diagnosis in our patient, it is important to consider other factors, such as cognitive overload of physicians. As demands for quality control, managing concurrent alerts, and notification fatigue in electronic health records continue to rise, cognitive overload and subsequent cognitive blindness can set in [13, 14, 15]. In the setting of our case of PAI, this possibly led to the continued anchoring on the diagnosis and treatment of gastritis and gastrointestinal‐related issues. Thus, it is essential that physicians and healthcare systems be wary of cognitive load and biases and recognize when a patient requires a reassessment to avoid the dangers of delays in diagnosis.

In 2013, a European Expert Consensus Statement was published, which recommended that all patients presenting with hypotension, vomiting, and/or diarrhea of unknown etiology should be clinically evaluated for PAI [16].

### 4.5 Literature Review

We conducted a focused literature review of the published English literature with key words of “addison's disease”, “primary adrenal insufficiency”, and “delayed diagnosis” in the databases Google Scholar, Pubmed, and Open Evidence AI with a goal of identifying common themes and factors contributing to the delay in diagnosis of PAI. The review revealed several cases of delayed PAI diagnosis and a multitude of potential factors that hinder timely diagnosis and treatment. The most commonly reported contributors include nonspecific diagnosis, slow rate of symptom onset, misinterpretation or absence of biochemical abnormalities, misdiagnosis, other misleading diagnoses, and medications as potential culprits. Cases of nonspecific presentation, similar to the case presented here, typically appear as gradual onset with generalized symptoms, such as malaise and gastrointestinal distress [17, 18]. Presence of lab abnormalities, such as hyponatremia, is commonly attributed to external factors, such as diet or emesis, or other diagnoses first, such as primary polydipsia [18]. Other classic electrolyte abnormalities, such as hypokalemia or hypoglycemia, may not be persistently present, as demonstrated in our case. Other physical exam signs associated with PAI, such as hyperpigmentation, may not be present, which then precludes PAI from being added to the differential. However, it should be noted that hyperpigmentation is observed in 94%–95% of cases of PAI, although the degree and spread of skin involvement widely varies, and thus may easily be missed on examination [2].

**FIGURE 3:**
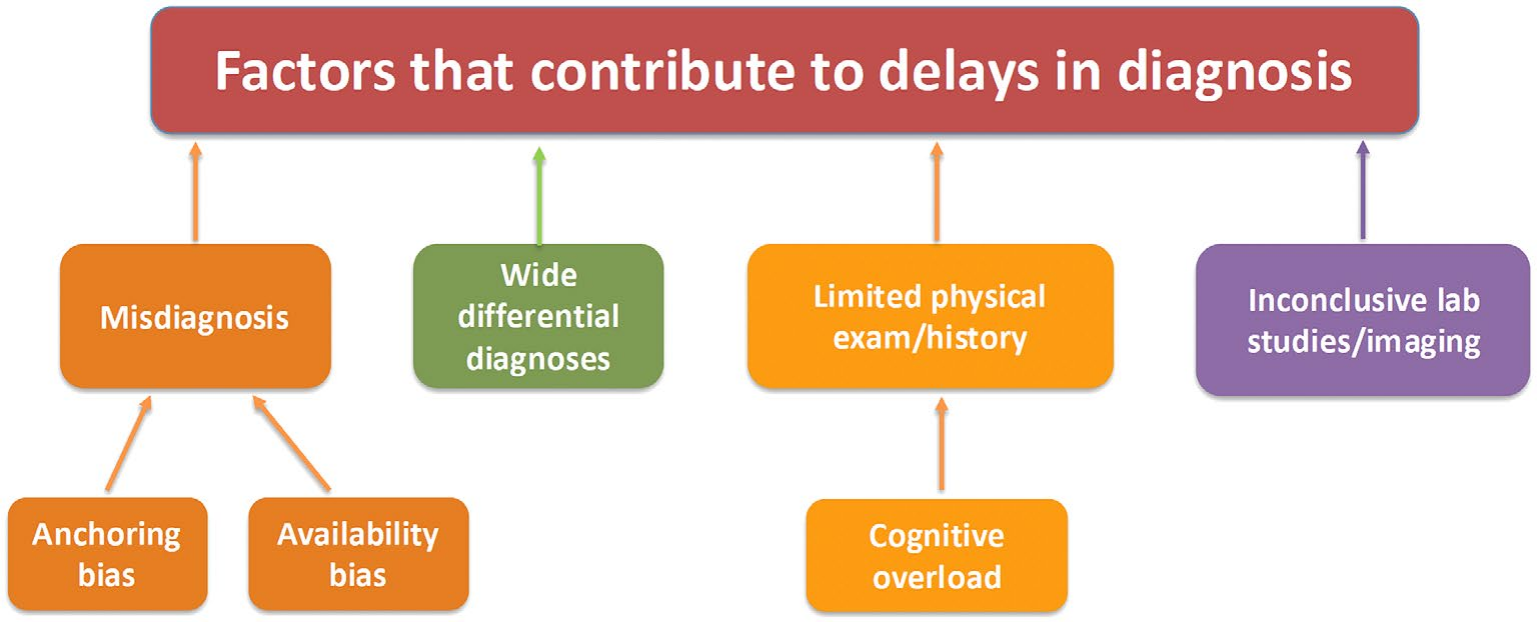
Flowchart representing how various factors can lead to delays in diagnosis of diseases such as Addison's disease.

In addition, patients are often initially misdiagnosed. Papierska and Rabijewski found that the most common initial diagnosis made for patients who present with nonspecific signs of PAI, such as loss of appetite, was chronic gastric and duodenal ulcer disease, resulting in unnecessary long‐term proton‐pump inhibitor therapy and delay in PAI treatment [19]. Once PAI has been included in the differential, it is evaluated via an initial ACTH stimulation test. However, hospitalized patients may have comorbidities that decrease the utility of this test. Critically ill patients may have varying responses to the ACTH stimulation test due to an expected higher than normal baseline cortisol level in these patients. A critically ill patient who is found to have a cortisol level that is within normal limits may be suspected of adrenal insufficiency, indicating a need for further evaluation [20]. Other patients may also be receiving glucocorticoid therapy for other comorbidities, which may mask symptoms related to glucocorticoid deficiency such as volume depletion. However, in PAI, these patients will continue to be mineralocorticoid deficient, and thus, continue to have symptoms and signs such as salt cravings and hyperkalemia [21]. Figure 3 highlights the various factors that can contribute to delay in diagnosis.

## 5 Conclusion

While a detailed history and physical is crucial to every patient encounter, the continued presence of cases of late PAI diagnosis suggests that other factors may be contributing to the delay, such as cognitive overload as previously mentioned. Several abnormal clinical presentations have been reported, such as acute renal failure, pancytopenia with cardiac tamponade, and initial presentation in an 80‐year‐old [22, 23, 24]. These cases serve as a reminder of the systemic effects of PAI, which can lead to various presentations. It is essential that PAI be added to the differential for nonspecific and systemic symptomatic presentation, especially in patients with concomitant autoimmune disorders.

Our case highlights the various challenges that can play a role in missed or delayed diagnosis of PAI. Early inclusion of PAI among the differentials in the right setting is crucial to make the right diagnosis. It can help decrease the risk of development of adrenal crisis, other effects of the hormonal imbalance seen in PAI, healthcare costs, and most importantly, suffering for the patient. Increased education of missed signs of PAI, including abnormal and unresponsive vital signs and lab values, presence of autoimmune pathologies, thorough physical exam including skin exam, and awareness of the role of cognitive overload in delayed diagnoses, among other factors, may help improve early diagnosis of this condition.

## Author Contributions

**Ishita Bhattacharya:** conceptualization, project administration, software, writing – original draft, writing – review and editing. **Lisle Blackbourn:** conceptualization, supervision, writing – review and editing. **Manajyoti Yadav:** resources, supervision, writing – review and editing.

## Funding

The authors have nothing to report.

## Consent

Written informed consent was obtained from the patient to publish this report.

## Conflicts of Interest

The authors declare no conflicts of interest.

## Data Availability

The data that support the findings of this case are as described. Further data is available from the corresponding author upon reasonable request.
